# First Trimester Aneuploidy Screening Program for Preeclampsia Prediction in a Portuguese Obstetric Population

**DOI:** 10.1155/2014/435037

**Published:** 2014-05-28

**Authors:** Cláudia Teixeira, Eduardo Tejera, Helena Martins, António Tomé Pereira, Altamiro Costa-Pereira, Irene Rebelo

**Affiliations:** ^1^Department of Pathology, Porto Hospital Center, 4099-001 Porto, Portugal; ^2^Department of Health Information and Decision Sciences (CIDES), Faculty of Medicine, University of Porto, 4200-450 Porto, Portugal; ^3^Institute for Molecular and Cell Biology (IBMC), University of Porto, 4150-180 Porto, Portugal; ^4^Department of Women, Porto Hospital Center, 4099-001 Porto, Portugal; ^5^Center for Research in Health Technologies and Information Systems (CINTESIS), Faculty of Medicine, University of Porto, 4200-450 Porto, Portugal; ^6^Department of Biochemistry, Faculty of Pharmacy, University of Porto, 4050-313 Porto, Portugal

## Abstract

*Objective*. To evaluate the performance of a first trimester aneuploidy screening program for preeclampsia (PE) prediction in a Portuguese obstetric population, when performed under routine clinical conditions. *Materials and Methods*. Retrospective cohort study of 5672 pregnant women who underwent routine first trimester aneuploidy screening in a Portuguese university hospital from January 2009 to June 2013. Logistic regression-based predictive models were developed for prediction of PE based on maternal characteristics, crown-rump length (CRL), nuchal translucency thickness (NT), and maternal serum levels of pregnancy-associated plasma protein-A (PAPP-A) and free beta-subunit of human chorionic gonadotropin (free **β**-hCG). *Results*. At a false-positive rate of 5/10%, the detection rate for early-onset (EO-PE) and late-onset (LO-PE) PE was 31.4/45.7% and 29.5/35.2%, respectively. Although both forms of PE were associated with decreased PAPP-A, logistic regression analysis revealed significant contributions from maternal factors, free **β**-hCG, CRL, and NT, but not PAPP-A, for prediction of PE. *Conclusion*. Our findings support that both clinical forms of EO-PE and LO-PE can be predicted using a combination of maternal history and biomarkers assessed at first trimester aneuploidy screening. However, detection rates were modest, suggesting that models need to be improved with additional markers not included in the current aneuploidy screening programs.

## 1. Introduction

Preeclampsia (PE) is a prevalent clinical entity in pregnancy, which is responsible for substantial maternal-fetal morbidity and mortality [1–4]. Prediction of PE could offer a window of opportunity for intervention during pregnancy, making it potentially possible to prevent adverse obstetric and neonatal outcomes.

In the United Kingdom, the National Institute for Health and Clinical Excellence (NICE) has delivered clinical guidelines recommending the evaluation of maternal risk factors for PE at the first prenatal visit for all pregnant women [5]. However, screening for PE based only on maternal history has shown to be insufficient [6].

In this context, measurement in early pregnancy of a variety of markers implicated in the pathophysiology of PE has been proposed to predict its development. These included tests for aneuploidy screening, renal and endothelial dysfunction, oxidative stress, and fetal-derived products [7]. Because any single biomarker is unlikely to be effective in prediction of the onset of a disorder as heterogeneous as PE, it is under investigation which combinations of tests, such as ultrasound and serum markers, would raise the effectiveness of history and physical-based screening [7].

The accuracy of models for PE prediction, either maternal history-based or combined with biomarkers, is unknown in the Portuguese population. Therefore, we aim to evaluate the performance of a first trimester aneuploidy screening program for preeclampsia prediction in a Portuguese obstetric population, when performed under routine clinical conditions.

## 2. Materials and Methods

### 2.1. Design, Setting, and Participants

**Figure 1:**
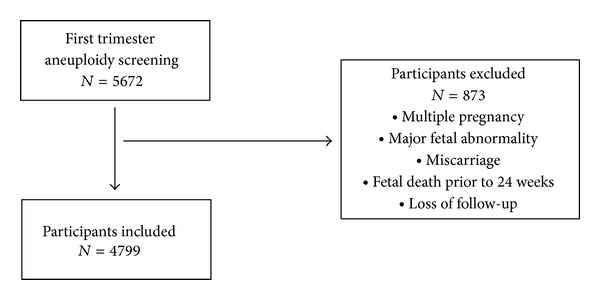
Flow chart of the study population.

This is a retrospective cohort study of 5672 pregnant women who underwent routine first trimester aneuploidy screening in a Portuguese university hospital (Centro Hospitalar do Porto) from January 2009 to June 2013. All singleton pregnancies between 9 weeks and 0 days and 13 weeks and 6 days of gestation were considered for inclusion in the study. Cases of multiple pregnancy, major fetal chromosomal or structural abnormalities, miscarriage, fetal death prior to 24 weeks, or loss of follow-up were excluded (*n* = 873) (Figure 1). All participants were followed from first trimester combined aneuploidy screening until delivery. The study protocol has been approved by the local ethics committee and institutional review boards.

### 2.2. Maternal Evaluation

Combined screening for aneuploidies was performed between 9 weeks and 0 days and 13 weeks and 6 days of gestation (*n* = 4799). Participants were asked to provide information on age, ethnicity, method of conception, weight, smoking status, chronic conditions, parity and previous pregnancy complications. Blood samples were collected and maternal serum pregnancy-associated plasma protein-A (PAPP-A) and free beta-subunit of human chorionic gonadotropin (free *β*-hCG) were measured using routine automated analyzers. During the study period, the analytical platform used by the clinical chemistry laboratory for measuring these biochemical markers was changed from IMMULITE 2000 system (*n* = 1634) to DELFIA XPRESS analyzer (*n* = 3165); this event was taken into consideration in the statistical analysis of data. An ultrasound examination was performed between 11 weeks and 0 days and 13 weeks and 6 days of gestation, including the measurement of crown-rump length (CRL) and nuchal translucency thickness (NT); gestational age was estimated on the basis of CRL measurements. These clinical data were systematically collected into an integrated electronic form in order to perform combined first trimester risk assessment.

### 2.3. Outcome Measures

Data on pregnancy outcome were collected from maternal and pediatric records. PE cases were defined by the new onset of hypertension (>140/90 mmHg) developed after 20 weeks of gestation in a woman with previously normal blood pressure, associated by coexisting significant proteinuria, according to the definition of the American College of Obstetricians and Gynecologists (ACOG) [3]. Chronic hypertension cases were defined as known high blood pressure before conception or new onset of hypertension before 20 weeks of gestation [3]. In the cases in which PE was superimposed on chronic hypertension, there was significant proteinuria development after 20 weeks of gestation in women with known chronic hypertension [3]. Cases of new onset of hypertension after 20 weeks of gestation in the absence of accompanying proteinuria were considered as gestational hypertension [3]. These outcome diagnoses were made by the treating physician and registered in maternal records at hospital discharge. Preeclampsia cases were classified as early-onset (EO-PE) or late-onset (LO-PE), depending on when findings first become apparent, before or after 34 weeks of gestation. We also included obstetric and neonatal outcomes in our analysis, such as gestational age at delivery, delivery by cesarean section, stillbirth occurrence, and birth weight. The adopted definition of low birth weight (LBW) was birth weight below 2500 grams.

### 2.4. Statistical Analysis

A descriptive analysis of maternal characteristics was conducted, separating the unaffected group from the women affected by preeclampsia according to their PE status, as described in the previous section. The maternal weight, PAPP-A, and *β*-hCG were expressed as multiples of the median (MoM) and log transformed for logistic regression analysis. Considering that two distinct analytical platforms were used for PAPP-A and *β*-hCG measurement, we adjusted these differences by performing the MoM transformation with the median values of the samples of both assays. The MoM values distribution obtained by this procedure were not statistically different when compared by analytical method using ANOVA analysis. We performed Mann-Whitney *U* Test and Pearson *χ* ^2^ for single categorical and quantitative variables analysis across groups. Moreover, the Kruskal-Wallis test was also used to compare the quantitative variables across multiples groups with subsequent pairwise analysis.

ROC (Receiver Operating Characteristic) analysis was performed to evaluate models performances and estimate predictive values, which were presented as the estimated detection rate (DR) at fixed false-positive rate (FPR) of 5% and 10%. All the binomial logistic models were obtained using stepwise backward algorithm for variable selection and a *P*-value cutoff of 0.05. We also presented the Nagelkerke *R* ^2^ for each model.

The statistical software package SPSS 21.0 [8] was used for data analyses.

## 3. Results and Discussion

### 3.1. Results

First trimester aneuploidy screening was carried out in 5672 pregnant women between January 2009 and June 2013. We excluded 873 cases because of missing outcome data (*n* = 715) or pregnancies resulting in miscarriage, fetal death prior to 24 weeks, or major fetal chromosomal or structural abnormalities (*n* = 158).

In the remaining 4799 cases, 140 developed PE (2.9%) and 4659 were pregnancies unaffected by PE. In the PE group, 35 (25%) developed early-onset PE and 105 (75%) developed late-onset PE. Biomarkers included in first trimester combined aneuploidy screening were available in all cases. A descriptive analysis of maternal characteristics, aneuploidy screening biomarkers results, and pregnancy outcomes is presented in Table 1.

In the PE group, compared to unaffected pregnancies, there was a higher median maternal age and weight as well as a higher prevalence of nulliparous women and history of chronic hypertension or diabetes mellitus. There were no significant differences in maternal ethnic origin, smoking habits, type of conception and infant gender between groups. In both the EO-PE and LO-PE groups PAPP-A were lower compared to unaffected pregnancies; there were no significant differences in free *β*-hCG, CRL, and NT. Median gestational age at delivery was lower in PE group compared to unaffected pregnancies and in EO-PE group compared to LO-PE group. Delivery by cesarean section was carried out in 70.5% of PE cases compared to 34.1% in pregnancies unaffected by PE. There was a lower median birth weight in PE group compared to unaffected pregnancies and in EO-PE group compared to LO-PE group, as well as a higher prevalence of LBW.

Although EO-PE and LO-PE were associated with decreased PAPP-A (0.93 MoM and 0.85 MoM), logistic regression analysis demonstrated that there were significant contributions from maternal factors, free *β*-hCG, CRL and NT, but not PAPP-A, for prediction of PE (Table 2). The *R* ^2^ values obtained were 10.0%, 9.4%, and 10.3% for PE, EO-PE and LO-PE, respectively. As expected, maternal history of PE, chronic hypertension, or diabetes mellitus contributed to the increase of the risk of PE, even when the last was not found significant for early expression of the disease. Similarly, higher maternal age and weight as well as nulliparous condition were also significant risk factors for PE.

**Figure 2:**
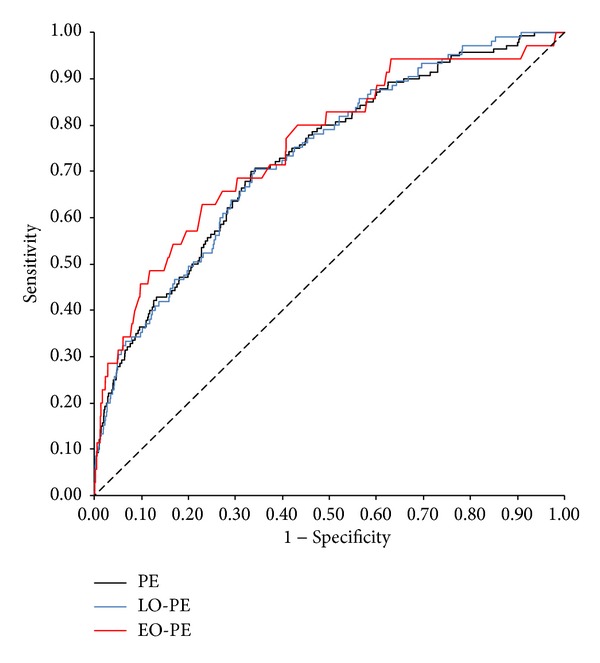
ROC curves of logistic regression models for prediction of PE, EO-PE, and LO-PE by maternal characteristics and aneuploidy screening biomarkers. PE, preeclampsia; EO-PE, early-onset preeclampsia; LO-PE, late-onset preeclampsia.

Our logistic regression models for PE prediction estimate that 27.9%, 31.4%, and 29.5% of PE, EO-PE, and LO-PE cases, respectively, could be detected with a 5% false-positive rate (Table 3). The model for EO-PE prediction presented the best performance (Figure 2), with detection rates of 31.4% and 45.7% at false-positive rates of 5% and 10%.

**Figure 3:**
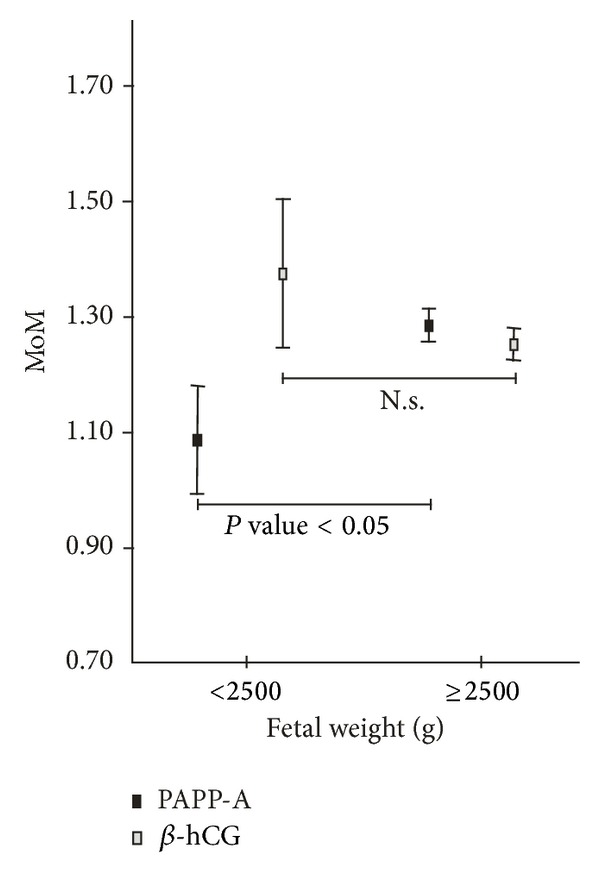
Variation of PAPP-A and *β*-hCG according to fetal weight at delivery (LBW).

A univariate analysis of PAPP-A and free *β*-hCG according to fetal weight at delivery shows lower median PAPP-A in LBW group but no significant differences of free *β*-hCG (Figure 3). Moreover, when we considered a logistic regression model for LBW, including all the previous variables, the final obtained model is 1.886 − 1.013 (if chronic hypertension) + 0.658 (if caucasian) − 0.570 (if smoker) + 0.196 (if multiparous) + 1.155 ∗ PAPP-A (MoM, Log) + 3.381 ∗ Maternal Weight (MoM, Log). This suggests that, unlike in PE prediction models, PAPP-A is related to LBW when other variables are included in a logistic regression model.

### 3.2. Discussion

Our cohort study of 4799 pregnant women who underwent routine first trimester aneuploidy screening in a Portuguese university hospital found a prevalence of PE of 2.9%, which is consistent with the reported epidemiologic data available in the literature [1–4, 9–11].

Our study provides evidence that both clinical forms of EO-PE and LO-PE can be predicted using a combination of maternal history and biomarkers assessed at first trimester aneuploidy screening, in agreement with previous publications [9–15]. As expected, regression models applied for prediction of the two forms of PE differed regarding which variables were included and performance achieved for each clinical form. However, these detection rates were modest, suggesting that models need to be improved with new information.

At false-positive rates of 5% and 10%, the detection rates for EO-PE were 31.4% and 45.7%, respectively, which are close to similar reported models [10, 12, 15]. Though EO-PE prediction presented a better performance compared to LO-PE, we must notice that its regression model only includes maternal clinical data, excluding biochemical and ultrasound markers. On the other hand, for LO-PE and overall PE prediction, logistic regression analysis revealed significant contributions from maternal factors, free *β*-hCG, CRL, and NT, but not from PAPP-A.

Although EO-PE and LO-PE were associated with decreased PAPP-A in the univariate analysis, this biomarker was not included in any of the logistic models for PE prediction. This could mean that the inclusion of PAPP-A in the models did not add further significant information to the one already provided by the others variables combined, hence the potential added value of PAPP-A measurement could be virtually negligible when used in combination with other biomarkers. These results suggest that a combination of free *β*-hCG, NT, and CRL could be more useful in PE prediction than PAPP-A alone. Our findings are supported by other studies [11, 16, 17] that also found a significant association between PE and PAPP-A that lost its significance when combined with others biomarkers and therefore did not contribute to PE prediction. Moreover, PAPP-A was significantly related to LBW, unlike free *β*-hCG, NT, and CRL; previous publications have shown inconsistent results regarding the association of birth weight with these biomarkers, as some studies reported a significant correlation [18, 19] and others did not [14, 20, 21].

Our study presents some limitations related to its retrospective nature. First, serum measurement of PAPP-A and *β*-hCG was not performed by the same assay method for all participants due to change of the analytical platform used by the clinical chemistry laboratory during the study period; however, this event was taken into consideration in the statistical analysis of data. Additionally, although several studies [9–11, 15] have shown that mean arterial pressure (MAP) is an important predictive variable for PE, data on maternal arterial pressure at first trimester screening were not available in clinical records. Furthermore, diagnosis of PE cases was made by the treating physician, which could constitute a potential source of bias. Nevertheless, it is the first study of its kind conducted under routine clinical conditions in a Portuguese obstetric population, reflecting the reality of nearly five years of performing a first trimester prenatal screening program.

As the traditional approach for PE screening based only on maternal demographic characteristics and medical history has shown to be insufficient [6], it is under investigation which combinations of markers would improve the performance of history-based screening. Ideal markers for PE screening should be easily measured and integrated within routine testing currently used as a part of prenatal screening. Furthermore, it would be helpful to integrate PE screening into existing analytical platforms to reduce costs, equipment, and human resources. Moreover, those markers should predict the risk in the first trimester of pregnancy, thus creating a wide window of opportunity to implement preventive or prophylactic treatment strategies which may facilitate normal placental development [22]. In this regard, biomarkers measured concurrently with testing for aneuploidies meet these requirements.

This study was conducted in a large unselected population under routine clinical care conditions, which supports the idea that screening for PE is feasible in obstetric populations with low* a priori* risk. However, our results suggest that the single inclusion of biomarkers currently used for aneuploidy screening in the prediction models for PE cannot achieve satisfactory detection rates and predictive values. Nevertheless, first trimester combined aneuploidy screening could be improved by inclusion of other biomarkers implicated in the pathophysiology of PE. Recent evidence suggests that serum placental growth factor (PlGF) and uterine artery pulsatility index (UtA Doppler) can be successfully included in preeclampsia prediction models with promising results [9–13, 15]. Although those results may be encouraging, it is difficult to achieve generalizable conclusions and standardized cut-points at specific gestational ages due to divergent study designs, population characteristics, and statistical approaches. Therefore, performance of PE screening should be validated in further large prospective studies.

Although the performance of such approach in Portuguese population is unknown, we believe that screening for PE could be successfully incorporated into routine prenatal care for assessment of patient-specific risk for PE and therefore offer a window of opportunity for intervention in early pregnancy.

## 4. Conclusion

Our findings support that both clinical forms of EO-PE and LO-PE can be predicted using a combination of maternal history and biomarkers assessed at first trimester aneuploidy screening. However, detection rates were modest, suggesting that the models need to be improved with additional markers not included in current aneuploidy screening programs.

## Figures and Tables

**Table 1:** Demographic characteristics of the study population.

Variable	Unaffected pregnancy (*n* = 4659)	PE (*n* = 140)	EO-PE (*n* = 35)	LO-PE (*n* = 105)
Maternal age, years, median (IQR)^∗a^	29.9 (25.8–33.0)	31.0 (27.7–33.6)	30.0 (25.0–34.9)	31.0 (28.0–33.0)
Maternal weight, median (IQR)				
Kg^∗a^	63.5 (57.0–72.0)	70.0 (60.9–82)	73.0 (64.5–82.0)	69.3 (60.6–83.4)
MoM^∗a^	0.99 (0.89–1.13)	1.10 (0.95–1.28)	1.14 (1.01–1.28)	1.07 (0.94–1.30)
Ethnicity, *n* (%)				
White	4529 (97.2)	138 (98.6)	34 (97.1)	104 (99.0)
Black	82 (1.8)	1 (0.7)	1 (2.9)	0 (0.0)
Other	48 (1.0)	1 (0.7)	0 (0.0)	1 (1.0)
Nulliparous, *n* (%)^b^	2843 (61.0)	98 (70.0)	27 (77.1)	71 (67.6)
Medical history, *n* (%)				
Chronic hypertension^b^	104 (2.2)	12 (8.6)	5 (14.3)	7 (6.7)
Renal disease	5 (0.1)	1 (0.7)	1 (2.9)	0 (0.0)
Diabetes mellitus^b^	47 (1.0)	9 (6.4)	1 (2.9)	8 (7.6)
Smoking during pregnancy, *n* (%)	975 (20.9)	21 (15.0)	6 (17.1)	15 (14.3)
Spontaneous conception, *n* (%)	4486 (96.3)	130 (92.9)	33 (94.3)	97 (92.4)
Ultrasound markers, median (IQR)				
CRL, mm	62.9 (56–70)	64.5 (58–70)	63.2 (56–68.9)	65.0 (59–70.5)
NT, mm	1.5 (1.2–1.8)	1.5 (1.2–1.8)	1.5 (1.2–1.9)	1.5 (1.1–1.8)
Maternal serum, median (IQR)				
PAPP-A, MoM^∗a^	1.01 (0.63–1.60)	0.85 (0.56–1.35)	0.93 (0.33–1.39)	0.85 (0.58–1.33)
Free *β*-hCG, MoM	1.00 (0.66–1.54)	1.10 (0.66–1.64)	0.93 (0.53–1.38)	1.17 (0.70–1.76)
Obstetric outcomes				
Gestational hypertension, *n* (%)	57 (1.2)	0 (0.0)	0 (0.0)	0 (0.0)
Cesarean section, *n* (%)^b^	1589 (34.1)	98 (70.5)	31 (88.6)	67 (64.4)
Gestational age at delivery, weeks, median (IQR)^∗a^	39 (38–40)	37 (35–38)	34 (29–36)	37 (36–39)
Neonatal outcomes				
Male, *n* (%)	2367 (50.8)	67 (48.2)	19 (54.3)	48 (46.2)
Stillbirth, *n* (%)	14 (0.3)	2 (1.4)	2 (5.7)	0 (0.0)
Birth weight, g, median (IQR)^∗a^	3165 (2873–3440)	2670 (2150–3055)	1910 (1050–2440)	2830 (2481–3158)
LBW, *n* (%)^b^	354 (7.6)	55 (39.6)	27 (77.1)	28 (26.9)

PE: preeclampsia; EO-PE: early-onset preeclampsia; LO-PE: late-onset preeclampsia; IQR: interquartile range; CRL: crown-rump length; NT: nuchal translucency thickness; *β*-hCG: beta-subunit of human chorionic gonadotropin; PAPP-A: pregnancy-associated plasma protein-A; MoM: multiple of the median; LBW: low birth weight. Significant comparisons between unaffected pregnancies and preeclampsia cases (*P* < 0.05) using: *Mann-Whitney *U* test, ^a^Kruskal-Wallis test, and ^b^Pearson *χ* ^2^.

**Table 2:** Logistic regression models for prediction of PE, EO-PE and LO-PE by maternal characteristics and aneuploidy screening biomarkers.

Variable	PE		EO-PE		LO-PE	
	*B*	*P*-value	*B*	*P*-value	*B*	*P*-value
Chronic hypertension [if true]	0.870	0.014	1.519	0.004	0.050	0.012
Diabetes mellitus [if true]	1.428	0.000			1.649	0.000
Parity [if multiparous]	−0.787	0.000	−1.201	0.008	−0.623	0.008
History of PE [if true]	3.952	0.000	3.201	0.001	3.668	0.000
Maternal age	0.039	0.026				
Maternal weight (MoM, log)	6.159	0.000	7.108	0.000	5.834	0.000
CRL	0.027	0.010			0.036	0.003
NT	−0.483	0.036			−0.592	0.026
Free *β*-hCG (MoM, log)	0.766	0.018			1.046	0.004
Constant	−5.723	0.000	−4.951	0.000	−8.248	0.000

Note: Missing values or any variable not included in the table indicate that those variables were not selected for the final regression models by absence of statistical significance. PE: preeclampsia; EO-PE: early-onset preeclampsia; LO-PE: late-onset preeclampsia.

**Table 3:** Detection rates and ROC results of logistic regression models for prediction of PE, EO-PE, and LO-PE by maternal characteristics and aneuploidy screening biomarkers.

Variable	ROC AUC	DR (FPR = 5%)	DR (FPR = 10%)
PE	0.732	27.9	36.4
EO-PE	0.754	31.4	45.7
LO-PE	0.734	29.5	35.2

PE: preeclampsia; EO-PE: early-onset preeclampsia; LO-PE: late-onset preeclampsia; AUC: area under the curve; DR: detection rate; FPR: false-positive rate.
